# From Single Molecule Fluctuations to Muscle Contraction: A Brownian Model of A.F. Huxley's Hypotheses

**DOI:** 10.1371/journal.pone.0040042

**Published:** 2012-07-16

**Authors:** Lorenzo Marcucci, Toshio Yanagida

**Affiliations:** 1 Graduate School of Frontier Biosciences, Osaka University, Suita, Osaka, Japan; 2 Center for Information and Neural Network, Suita, Osaka, Japan; 3 Quantitative Biology Center, RIKEN, Suita, Osaka, Japan; University of Zurich, Switzerland

## Abstract

Muscular force generation in response to external stimuli is the result of thermally fluctuating, cyclical interactions between myosin and actin, which together form the actomyosin complex. Normally, these fluctuations are modelled using transition rate functions that are based on muscle fiber behaviour, in a phenomenological fashion. However, such a basis reduces the predictive power of these models. As an alternative, we propose a model which uses direct single molecule observations of actomyosin fluctuations reported in the literature. We precisely estimate the actomyosin potential bias and use diffusion theory to obtain a Brownian ratchet model that reproduces the complete cross-bridge cycle. The model is validated by simulating several macroscopic experimental conditions, while its interpretation is compatible with two different force-generating scenarios.

## Introduction

Forty years ago, A.F. Huxley and R.M. Simmons [1] reported the behaviour of skeletal muscle fiber by observing near instantaneous force changes following fast and small length perturbations. Accordingly, Huxley and Simmons proposed that the force is generated from an actomyosin complex that exists in (at least) two stable but different force-generating conformations, or states, that oscillate between one another in response to external perturbations. This theory has since been analysed by several studies examining muscle structure [2]–[4], macroscopic muscle fiber behaviour [5]–[9], and single molecule experiments (SME) [10]–[13] including recent SME studies that have directly observed the oscillations [14], [15]. In their same seminal paper, Huxley and Simmons proposed a mathematical model that described an explicit relationship between the probability of the actomyosin state and the external perturbation applied. That model has since been refined by several works and extended to the entire cross-bridge cycle [5], [16]–[20].

**Figure 1:**
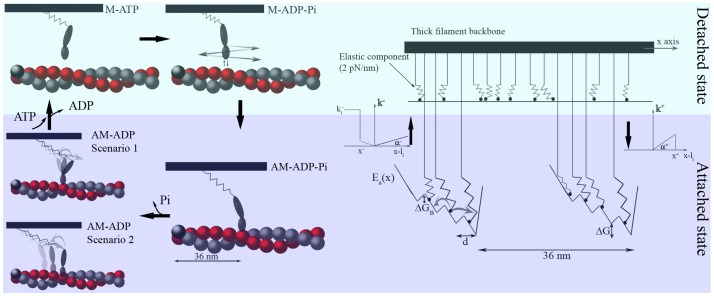
Cross-bridge cycle (left) and half-sarcomere model (right). Left side: Four states, two detached (upper) and two attached (lower), are considered. In the force generating step, the actomyosin complex oscillates between stable states either by rotating the lever arm domain (scenario 1) or by sliding the myosin head along the actin filament (scenario 2). Right side: Mechanical representation of the half-sarcomere as many parallel myosin heads. In the detached state, thermal fluctuations by a myosin head are constrained only by an asymmetric elastic element [29]; the absence of other interactions is represented by the flat energy landscape. In the attached state, the head also experiences an actomyosin complex energy landscape, 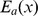, which has a periodicity of 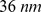 and four minima or stable states (see text). The jump process between attached and detached states is driven by the rate functions 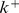 and 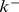 (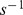) to generate a flashing Brownian ratchet.

As informative as these contemporary models are, they are limited due to the lack of direct information on the state transition dynamics. While they introduce an explicit definition of the energy in each state, they define the transition between states as rate functions with *ad-hoc* dependencies on the chemical reaction coordinate or base the transitions on hidden assumptions of the actomyosin potential energy. In other words, actomyosin properties are deduced by fitting the macroscopic behaviour of the muscle fiber even though the goal of the model is to interpret the muscle behaviour from the actomyosin properties. We believe that this approach reduces the predictive power of such models because the oscillatory behaviour of the actomyosin complex is a fundamental property of muscle function such that its description cannot be restricted to phenomenological rate functions. Therefore, we here describe an alternate model based on diffusion that considers the entire Lymn-Taylor cycle (Fig. 1). Although a diffusional approach for the attachment-detachment process is not feasible because of insufficient experimental data for skeletal myosin, configuration changes in the actomyosin complex can be described by thermal diffusion if the periodic potential is properly designed for there to be a clear relationship between the chemical configurations and the external force applied. Thus, we approach the problem by defining a diffusional model using SME data 14,15 and validate our hypotheses by simulating experimental macroscopic muscle fiber data. This new approach (see also [21]–[23]), and its future application to the attachment-detachment process, aims to reduce the degree of freedom in the parameters to more accurately explain muscle behaviour by using SME data.

## Materials and Methods

### Cross-bridge Cycle and Model Description

In its most basic form, the cross-bridge cycle can be described as a four-state model with two detached and two attached states (upper and lower parts of Fig. 1, respectively). The left side of figure 1 shows a detached myosin head that initially has a low affinity for the actin filament (M-ATP). The myosin next hydrolyses ATP to generate a high energetic state (M-ADP-Pi). Brownian fluctuations then allow the myosin to bind to the preferred actin-binding position to form the actomyosin complex AM-ADP-Pi. This attachment is probably driven by the “Brownian search-and-catch mechanism”, which assumes that the affinity for actin increases when the myosin elastic element is stretched forward [24], [25]. Force generation stretches the elastic element even more so that the complex takes the AM-ADP state. Detachment occurs when a new ATP molecule substitutes for the exhausted ADP molecule (M-ATP) to relax the elastic element and begin a new cycle.

Oscillating sub-steps during the force generating (AM-ADP) state have been observed experimentally by attaching a single myosin II molecule to a large micro-needle and associating it with an actin bundle [14], [15]. This results in several 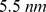 steps per ATP cycle that are biased in one direction, as predicted in [1]. The steps are conventionally explained by the lever arm theory: the long portion of the myosin head rotates to slide the actin and myosin filaments past each other while the globular portion remains firmly attached to the actin monomer (Scenario 1 in Fig. 1) [1], [17], [26], [27]. Despite that, the constant size and frequency of the steps observed experimentally may be generated by a different mechanism where the helical shape of the actin filament drives the sliding of the myosin head on different actin monomers to pull the thick filament (Scenario 2 in Fig. 1) [14], [15] and the rotation of the lever arm takes place only at the end of sliding during Pi release. This scenario has been recently supported by MD simulations of skeletal actomyosin in the rigor configuration [28]. In that work, the preferential direction of thermal fluctuations in the actomyosin complex was shown to arise without ATP consumption.

In both scenarios, the steps are generated by stable actomyosin states which mathematically correspond to several minima in the potential energy, 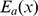, which describes the actomyosin interaction (Fig. 1, right). Thus, our model does not discriminate between the two scenarios and defines the energy potential using only SME data. Force is generated when the actomyosin complex fluctuates between stable states in a manner that allows the complex to adapt efficiently to different external stimuli. The key feature of the model is that mathematically these fluctuations are described as diffusional motions inside 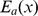. Therefore, we do not need AM-ADP-Pi to AM-ADP transition rate functions, nor do we need to consider the dependence of these functions on the head position when fitting the macroscopic behaviour of a contracting muscle. On the contrary, having 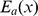 defined by SME data alone means we can use macroscopic behaviour to test the goodness of the shape of the potential.

To model the macroscopic response (see Text S1 for method), we assume 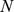 myosin heads are in parallel in a one-half sarcomere, and that several sarcomeres are in series and behave uniformly. The myosin heads are attached through an asymmetric elastic element [29] to a rigid backbone (thick filament, right side of Fig. 1) at uniformly spaced locations due to the incommensurability of the periodicity of the two filaments [30]. In order to maintain a clear chemo-mechanical relationship, we define a minimalistic model where the myosin can exist in only two different states, one force generating and one non-force generating (zero on average), which roughly relates to the attached and detached states, respectively. Transitions between these states are regulated by the stochastic variable 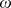 (pure number), which fluctuates between the values 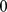 and 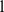.

In the detached state (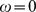), the myosin head is subjected to unbiased thermal fluctuations and to the force generated by the elastic element. The transition from M-ATP to M-ADP-Pi is a diffusion driven process that does not require a rate function. When myosin interacts with the actin filament (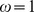), however, it is also subjected to the force generated by 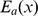. Each myosin molecule is considered an over-damped particle whose geometry is completely defined by its drag coefficient, 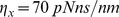 [31]. The environment in which the myosin moves is defined by the Boltzmann constant, 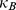, and the absolute temperature, 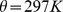. The motion of the 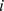-th myosin head leads to a flashing ratchet system with its dynamics described by the Langevin equation:
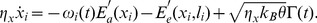
(1)where 
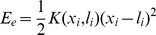
 is the asymmetric [29] elastic energy of the myosin globular portion, lever domain, and long tail in series. Stiffness is defined as



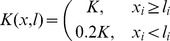
where 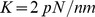 [17] and 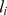 is the reference position on the backbone of the 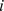-th head. By setting 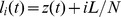, where 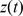 is the position of the backbone, continuous behaviour of the myofibril is ensured by having 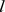 uniformly distributed over 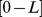 (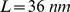). 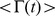 is a random term constrained by 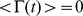 and 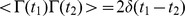 (white noise). Total force generated by the myosin heads is given by 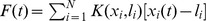.


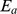
 is modeled as a piecewise linear, multi-stable potential with minima equally spaced by a distance that is compatible with the actin monomer diameter, 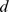, and a directional bias defined by 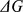. Although the potential is locally biased, it is flat on average, repeating itself every 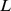, with each period containing six actin monomers (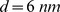).

Experimentally, the number of steps ranged from one to five at low micro-needle stiffness (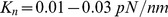 [14]) and one to four at high micro-needle stiffness (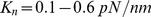 [15]). We impose that the myosin can interact with only four actin monomers, and attachment cannot occur in the region 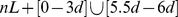, where 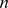 is an integer, a constraint that mimics steric effects in the actin double helix.

Since the potential is globally flat, net movement in the backbone will only occur by breaking the global equilibrium [23]. We introduce a *jump process* that constrains 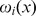 to follow the rate functions 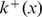 and 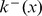 (described in Fig. 1) out of balanced equilibrium by setting 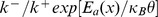. Referring to the Brownian search-and-catch mechanism originally proposed by A.F. Huxley in 1957 [25] and seen in Myosin VI [24], we introduce an attachment process where a detached myosin head searches for its preferred actin-binding site in the forward direction by thermal fluctuations. The detachment rate function 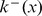 we use is a slightly modified version from the one proposed in [25] in order to consider the more detailed geometry of the energy potential.

### SME Simulations

**Figure 2:**
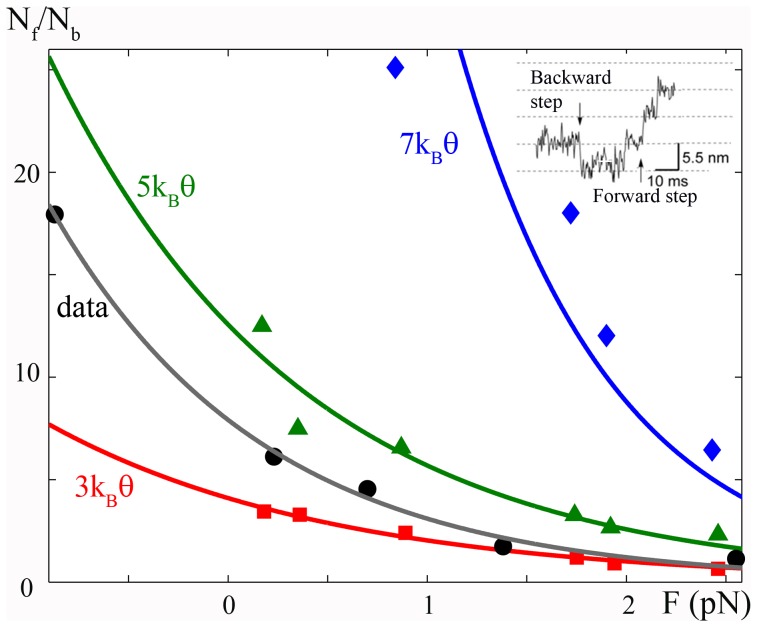
Bias of actomyosin energy. The diffusion of a particle linked to an external micro-needle in a periodic potential tilted by a constant bias, 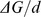, is simulated, and the ratio of forward, 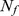, and backward, 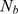, jumps for different 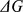 is reproduced and compared to the experimental data. Red squares, 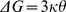; green triangles, 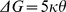; and blue rumble, 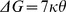. Black dots, experimental data from [15]. Continuous lines are the corresponding exponential fits. Simulations show that a bias higher than 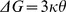 corresponds to the observed ratio. A value of 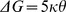 is chosen as most probable due to the boundary conditions (see text). Insert: Experimental oscillating sub-steps as expected from [1] during the attached state (see fig. S2). Figure adapted from [15].

To quantitatively define the energy bias for muscle in vivo, we simulated in vitro data from [14] and [15] (Fig. S1). Previously, a bias of 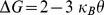 is obtained in [15] assuming the micro-needle applies a constant force, 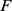, to the molecular motor during a jump and 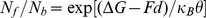 (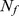 and 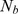 are the number of forward and backward jumps, respectively). Nevertheless, the drag coefficient, 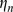, of the micro-needle was three orders of magnitude higher than that of the molecule, meaning the external system applies a force that varies with the instantaneous position of the myosin during a jump. Therefore, the previous estimation may underestimate the real 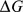. Consequently, we numerically explore the effect of 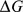 on 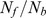 by simulating the micro-needle displacement for several 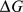 and by comparing the resulting 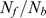 with the experimental data (Fig. 2). Because in those experiments only the S-1 portion of the myosin head was used, we imposed a symmetric molecular stiffness 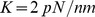 [17]. The micro-needle is also represented as an over-damped particle whose drag coefficient is 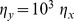 [32] and is maintained near its original position by stiffness 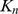 [15].

The set-up is described by the following system of stochastic differential equations:
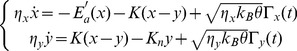
(2)where 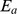 is the actomyosin potential shown in Fig. 1. Since the ratio of forward and backward jumps were analysed in [15], we impose the mean value of needle stiffness used in that experiment 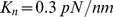. Three parameters are needed to define the potential: the energetic barrier, 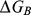, between two minima; the asymmetry of the potential, 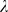, which is the ratio of the distance between an adjacent maximum and minimum and 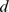; and the bias or energy difference, 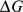, between two minima. We impose an energy barrier of 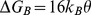, which is compatible with myosin II conformational changes that occur in less than one millisecond in the unloaded condition [33], as obtained by the exact solution of the first passage time, 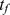, of a particle against a constant force 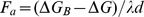 [31].

During a change of state in a multi-stable energy potential, the particle spends most of its time surmounting the energy barrier [34]. We suppose that the maximum of the barrier between two minima is displaced toward the forward minimum (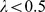), as in [1] where this distance (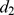 in Fig. S1) is zero. Numerically we cannot impose a zero distance, thus we do the following reasoning. The distance between the steps observed in [14], [15] was 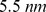. This should equal the distance between two stable states, or minima in the energy potential. Assuming 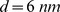, we set 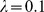 so that the distance between a minimum and the subsequent maximum (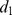 in Fig. S1), the portion of the potential that influences the step, is 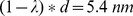. Then we simulated eq. (2) for different external forces based on the micro-needle position and different values of 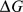. A typical simulated trace is seen in Fig. S2. This is comparable with the experimental trace shown in the inset of Fig. 2. The analysis shows that 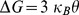 (red line) results in a 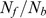 ratio that always underestimates the experimental value (grey line). On the other hand, if one assumes 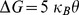 and 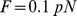, then the relation used in [15] should lead to 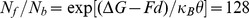, whereas our numerical results give a ratio one order of magnitude smaller, showing that the previous relation strongly underestimates the effect of the micro-needle size.

In order to minimize the number of parameters, we imposed some simplifications on our SME simulations. In particular, the myosin head is considered to be attached in the active state such that we ignore the rigor state. In other words, myosin always oscillates between attached stable states. This situation creates a boundary effect such that when the head reaches the last minimum it can only jump backward. Due to the bias of the potential, this means the longer the simulation, the lower the 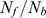 ratio. To overcome this problem, we simulated the trajectory of each particle until the lowest minimum is reached and then interrupted the computation. In the experimental protocol of [15], jumps were measured until the rigor state occurred, which likely resulted in underestimating the ratio with respect to our method. We therefore concluded 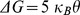 as the best estimate for the data and use this value to define the bias for the remainder of our analysis.

Summarizing the known and unknown parameters, the myosin stiffness, 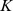, the drag coefficient, 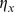, and the energetic barrier, 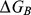, are known or accepted values from the literature, while the temperature, which defines 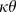, the needle stiffness, 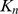, and the drag coefficient, 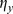, are known experimental values. The energy bias, 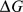, is the variable under analysis and is varied over a range of realistic values. Then, the unknown or free parameters that remain are the potential asymmetry 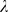 and the distance between minima 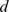. The product of these two values is chosen to satisfy the observed step length in the computational limit of our method (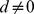).

### Model Simulations

One limit of the diffusional approach is the time required to perform a real-time (up to hundreds of milliseconds for the force velocity curve) simulation of the muscle system. The reason is that for equation (1) to have any statistical meaning, the time step of the simulation must be at least two orders of magnitude less than the characteristic time scale of the system. We therefore introduced the following constraints. We set 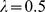 to increase the time step and 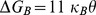 to reduce the energetic barrier maximum in the periodic potential. To evaluate the effects of these constraints, we compared the dwell times of a free particle to the two 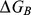 values, 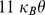 and 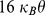, finding the lower energy barrier made the dwell time approximatively 150 times shorter. Consequently, we also increased the attachment and detachment rates in a way that maintains the relative velocity to approximate those seen in experiments.

Numerically, we imposed 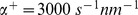, 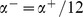, 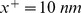, 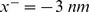, and 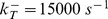 (see Fig. 1). Thus, when considering a distance 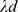, the maximum attachment rate is about 150 times faster than the realistic value of 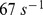 [20]. These values result in a simulated behaviour that is faster than that seen experimentally. For example, the simulated maximum velocity (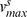) is 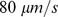, which is 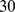 to 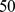 times faster than the experimental value (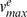) for frog muscle [33], [35]. Thus, we must slow the simulated processes by 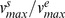 to obtain a corresponding quantity in real-time. To prove the feasibility of this approach, we simulated the rising phase curve assuming that all heads are initially detached in the isometric (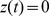) state, slowed the simulated curve by a factor of 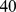 (an average of the two 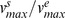 values), and compared it to that seen experimentally (Fig. S3), finding the two superimposed almost perfectly. Therefore, the attachment and detachment rates in our model are proportional to the velocity of contraction in a way compatible with the experimental results.

Summarizing, the protein drag coefficient, 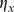, its stiffness in the stretched configuration, 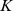, and the temperature (or 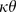) are known or accepted values. The potential periodicity, 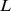, the uniform random distribution of proteins, the number of monomers (six, 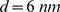), and the constraints for the four attachment sites, which relate to the helical shape of the actin filament, are either known values in the second scenario or are new experimental constraints for the first scenario. A potential bias, 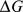, is also imposed by the experimental data (see previous sub-section). The number of proteins, 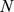, and the myosin filament drag coefficient, 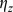, are scale parameters that do not affect the normalized results below. Protein stiffness in the compressed configuration, even if qualitatively imposed in accordance with recent experimental observations, is used to fit the data and must be considered a free parameter. The potential asymmetry, 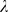, and maxima 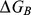 in the model simulation are also free parameters used to modify the time scale to make the calculation possible. Finally, the parameters needed to describe the attachment and detachment rates (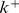 and 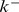, in 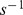), 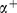, 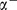, 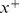, 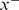, and 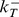, are free parameters whose values are chosen in accordance with 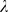 and 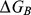 to satisfy a coherent rising phase and maximum velocity (see above).

A comparison with other macroscopic models is difficult to make due to differences in fundamental conditions and to the wide range of simulated data. Despite that, the number of free parameters is relatively low and mostly attributed to the attachment-detachment process.

## Results

**Figure 3:**
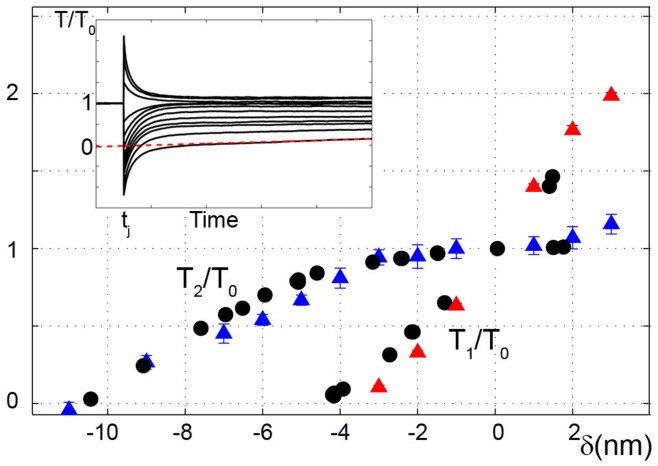
Fast tension recovery. Simulations and experimental data for a small and fast length step, 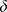, applied to a muscle fiber in isometric contraction. The simulated tensions after the imposed step, 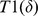 (red triangles), and after actomyosin re-equilibration, 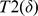 (blue triangles), are shown and compared to the experimental results (circles; data from [33]). All tensions are normalized with respect to isometric tension 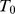. Simulated Tension vs. Time traces are shown in the insert at different 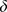, 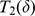 is estimated by the tangent method [41]. Mean values
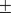
standard deviation over 11 trials are shown. Where error bars are not visible, errors are smaller than the symbol width.

**Figure 4:**
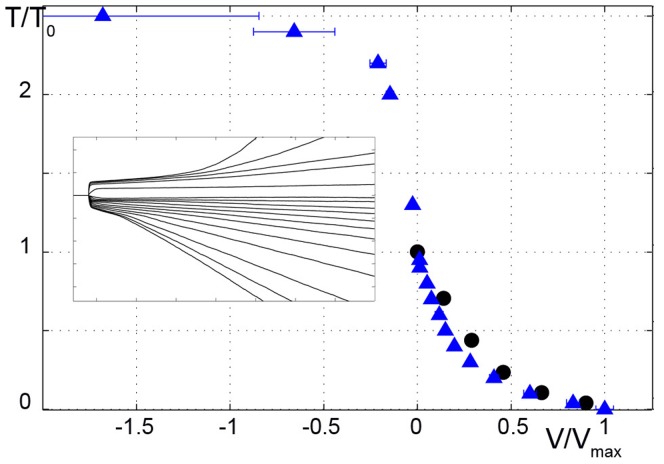
Velocity of contraction at different external loads. Applied tension is reduced or increased with respect to isometric tension at a given time. Simulated Tension vs. Time traces are shown in the insert. Tensions are normalized to the isometric tension, 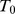. For different tensions, velocities are calculated from the linear portion of the shortening traces and normalized to the maximum velocity. The simulated velocities vs. tensions (blue triangles) are compared with experimental data (black dots) from [35]. In the concentric region (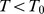), the simulations fit the experimental data well even if the velocity is underestimated in the central part (see text). Experimental data for the eccentric region (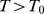) are not shown, but a qualitative correspondence can be observed with a plateau region at high tensions. Mean values
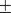
standard deviation over 11 trials are shown. Where error bars are not visible, errors are smaller than the symbol width.

We quantitatively tested our model for two classical skeletal muscle experiments: fast tension recovery after a small increment in isometric length (length clamp), and velocity of contraction against a constant load (load clamp). For the first case, a fast (microsecond) and small (few nanometers per half sarcomere) change in muscle length, 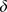, during isometric contraction resulted in typical tension transient behaviour (Fig. 3, inset). Initially, an almost instantaneous change from the isometric tension 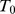 to tension 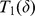 is seen. This is followed by a slower (milliseconds) tension recovery that plateaus at 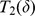. Over a longer time scale, a new population of myosins attach such that tension recovers to 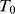. In the second case, a muscle fiber bears a constant load which generates a tension 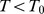 and contracts at a constant velocity that depends on the load in an hyperbolic manner (Fig. 4) [36].

The model can simulate the length clamp behaviour by using equation (1) and assuming:
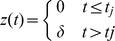
which experimentally describes a length change at time 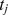. The force clamp behaviour is simulated by applying equation (1) and assuming



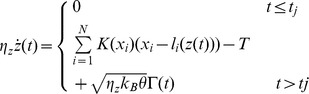
where the drag coefficient of the thick filament, 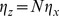, considers the total number of motors and 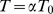 is the external tension, with 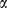 ranging from 0 to 2.5.

Simulations for both 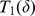 and 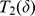 curves (Fig. 3) and the 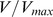 vs. 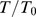 curve (Fig. 4) show very good fitting. The latter also shows qualitative fitting for the 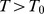 region.

**Figure 5:**
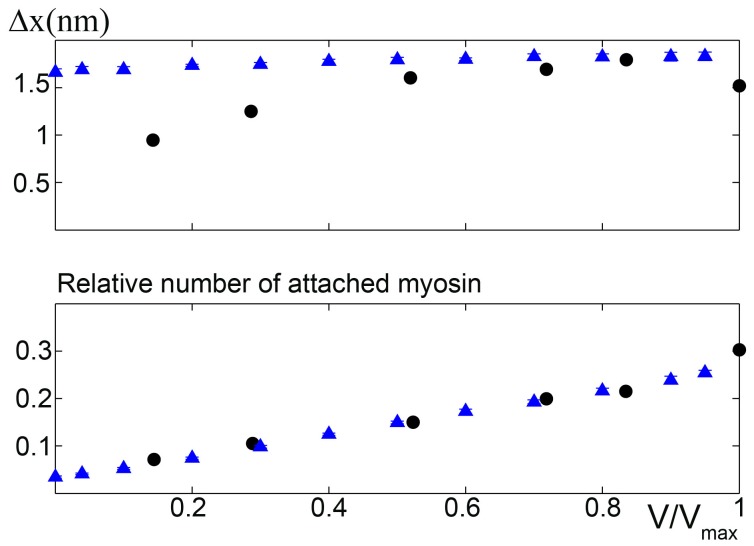
Microscopic behaviour of the model Upper: Simulated mean strain per motor during the steady state phase of isotonic contraction at different external tensions compared with experimental data from [8]. Mean strain is well fitted at high 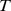, but overestimated at low 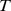, where the low number of attached motors may have compromised the experimental analysis, as observed in [8]. Lower: Relative number of attached motors at different loads during the steady state phase of isotonic contraction. Simulation, triangles; experimental data, circles. Mean values
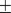
standard deviation over 11 trials are shown. Where error bars are not visible, errors are smaller than the symbol width.

To further investigate the model predictions, we simulated the number of attached myosins during isokinetic contraction under different external loads and compared the results with experimental data obtained from [8], where the molecular basis of the force velocity relationship was determined by X-ray interference and mechanical measurements on intact single cells. Assuming asymmetric stiffness, that study could only determine the number of stretched myosins, not all attached myosins. We simulated the average number of stretched myosins during the steady phase of an isotonic contraction at various 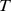, finding the data could be fit excellently without any supplemental hypothesis (Fig. 5, lower). Also, the predicted mean strain per motor is in very good agreement with the experimental data at high and medium forces (Fig. 5, upper). The different behaviour at low loads can be attributed to the low number of attached motors, which may have compromised the experimental analysis.

**Figure 6:**
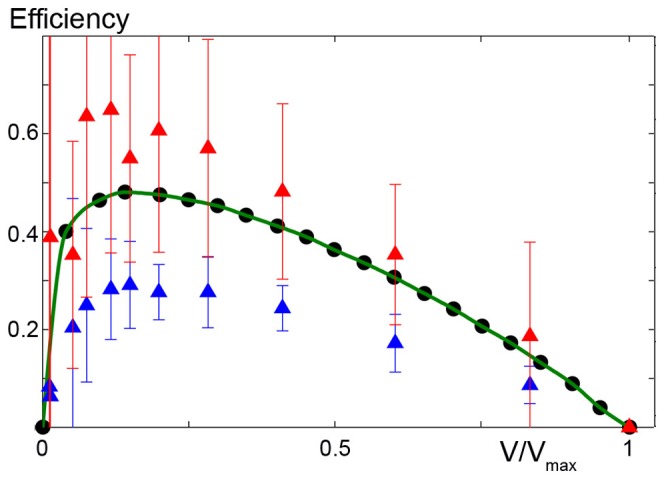
Predicted efficiency at different velocities Simulation of the efficiency of contraction, as predicted by the model compared to experimental data (circles from [38], green line). Efficiency is computed as tension times velocity divided by the chemical energy consumed [37] (triangles) and following the method described in [39] (squares). Mean values
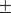
standard deviation over 11 trials are shown. Where error bars are not visible, errors are smaller than the symbol width.


Finally, we can conclude our model results are not due to inefficient cycling (only a small fraction of ATP energy being converted to work). Noting that each detachment process consumes one ATP molecule, we measured the efficiency of the flashing Brownian ratchet as the product of the force and the velocity of contraction divided by the total energy consumed [37]. Defining 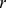 as the number of detaching cross-bridges per unit time, we can write 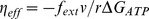. In Fig. 6, blue triangles show efficiency versus the velocity of contraction assuming 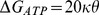. The maximum efficiency was more than 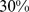, which is comparable with experimental values [38]. We observe that the model prediction is rather good even though it somewhat underestimates the efficiency. This is likely because we only consider two states to simplify the model. In other words, we ignore the condition where myosin heads attach and detach to the actin filament without generating force or consuming energy (weakly attached state). Incorporating this state should increase the efficiency. To estimate how much of an increase, we computed the physical upper bound of the efficiency produced by the ratchet according to the formula proposed in [39]:
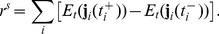
(3)


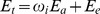
 is the total energy computed before 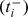 and after 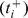 the 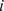-th jump, 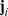. The summation is done for every jump that occurs per unit time in the region of steady shortening. The efficiency limit is thus given by 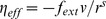 shown in Fig. 6 (squares). The experimental efficiency is between the two model efficiencies, indicating a more detailed description of the states can improve the fitting.

## Discussion

In this work, we have used diffusion theory to model muscle mechanics, defining some parameters using SME data and quantitatively simulating several macroscopic behaviours. The model satisfies two different hypotheses regarding the origin of the actomyosin stable states, which are often viewed as stable states of the lever arm (scenario 1). This interpretation gives a clear picture of the role of the lever arm structure in muscle contraction and is compatible with X-ray reflections during isotonic contractions that describe the regular arrangement of myosin motors on the backbone [7], [8]. However, if we assume scenario 2, where each minimum of the actomyosin potential energy corresponds to a given actin monomer on the thin filament, the role of the lever arm structure is more equivocal. That the actin diameter is about 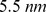 means the vertical distance between the backbone and different attachment sites in one half-pitch varies considerably due to the three-dimensional structure of the actin filament. Thus, we can assume that the orientation of the lever arm plays an important role for myosin reaching the binding sites. In our model, we assumed one-dimensional geometry for simplification. Nevertheless, we can still infer some aspects of the lever arm orientation at different minima. In the insets of fig. S4, we describe the distribution of the attached heads in the four subsequent minima in one half-pitch shown in Fig. 1 from the highest (d) to lowest (4d) minimum at different times of an unloaded force-clamp simulation. We can see that at time 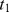 during an isometric contraction, all four minima are populated, but the central ones are more populated. As expected, at the end of phase 2 in the load-clamp simulation (time 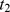), the lowest minimum (4d) is the most populated, while the first two minima populations are near zero. By associating each minimum a different lever arm orientation, the periodic distribution of the myosin head mass along the thin filament becomes very different such that the distribution can justify the different experimental X-ray reflection observations produced at times 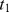 and 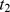 [7], [8]. Also, during isokinetic contraction (time 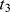), the population re-equilibrates toward a more homogeneous distribution, which is consistent with experimental observations.

It is worth noting that scenario 1 allows for more flexibility than scenario 2 when defining parameters such as the distance between minima. Nevertheless, the good fitting obtained shows that either scenario is consistent with the experimental data. Moreover, high speed AFM showed that a myosin V head undergoes brief translocation along actin by repeatedly detaching and attaching after Pi release, i.e., at the force generating state [40], giving creedance to scenario 2. To determine which of the two scenarios is the true source of force generation, more experimental evidence is needed.

One limit of our model is that the contraction velocity is slightly underestimated for mean values of applied force. Consequently there is an enhanced underestimation of the maximum power output 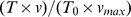 (Fig. S5). This limit is common to other macroscopic models [20], [35], and can be due to a low number of simulated attached heads in the central part of the force-velocity curve. The number of attached cross bridges can be increased by a higher attachment rate, but that would create an unrealistically fast rising phase during isometric contraction [35]. A way to resolve the problem is to hypothesize an attachment rate that depends on the velocity of the contraction in addition to the dependency on the position of the head [20]. Despite using a different physical description of force generation in the attached state (diffusion), our model shares with the above models the same chemical description of the attachment-detachment process (rate functions) because of a lack of experimental data. Thus, it is not surprising that our model suffers from underestimating the maximum power output. Resolving this problem would benefit from a description of the attachment-detachment process by incorporating diffusion into the energy landscape.

### Conclusions

Although the mechanics of muscle contraction have been well studied, a comprehensive model that reproduces at the same time all macroscopic behaviour is still lacking. Furthermore, one weakness in muscle modelling is that the same behaviour can be reproduced starting from different working models. We believe that this is in part because the description of the oscillatory behaviour of the actomyosin complex, which is generally accepted as a fundamental property of muscle function [1] and whose properties are becoming more accessible from SME, is often restricted to rate functions based on the macroscopic behaviour of muscle fibers.

To emphasize the importance of this oscillatory behaviour, we have applied a new approach to muscle modelling, where actomyosin complex dynamics are described by the diffusion of particles constrained within a defined energetic landscape that is described by SME data. Having inferred the bias of actomyosin energy, we showed the feasibility of this approach for quantitatively describing several macroscopic behaviours. Furthermore, the new approach reduces the number of free parameters and makes them constant rather than empirical functions that are based on chemical reaction coordinates. This model can potentially be a basis for future developments where parameters that describe the behaviour of single molecules can be built upon to understand all contraction mechanism details, such as how molecular cooperation leads to macroscopic behavior.

## Supporting Information

Figure S1 **SME set-up from** [**32**] **. An external system (microneedle) with a very high drag coefficient is linked to a multistable particle (attached skeletal myosin).** The motion of the particle is analysed through the position of the microneedle. A linear spring with symmetric stiffness links the two bodies.
(EPS)

Figure S2 **Example of a simulated microneedle trace based on Figure S1.** The myosin head is always in the attached configuration. The trace is comparable with the experimental results obtained in [15].
(EPS)

Figure S3
**Simulation of the isometric contraction.** The simulated rising phase is slowed by a factor of 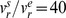 (blue trace). Simulations for 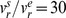 (green trace) and 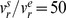 (yellow trace) are also shown. The slowed rising phase is in agreement with the experimental data (red trace from [42], the initial difference is due to the latency of force development and stretching of the tendons, which are not considered in the model).(EPS)

Figure S4
**Distribution of the attached myosin heads (insets) in the four minima of actomyosin energy** 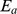 **during different phases of the unloaded force-clamp simulation (continuous line).** Scale in the insets is normalized to the number of attached heads.(EPS)

Figure S5
**Power output predicted by the model (blue triangles) and comparison with experimental data (black circles).** The lower velocity of contraction at intermediate tensions leads to an underestimation of the maximum power output. This drawback is common with other models and is related to the attachment-detachment process (see main text). Mean values
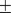
standard deviation over 11 trials are shown. Where error bars are not visible, errors are smaller than the symbol widths.(EPS)

Text S1 (PDF)

## References

[1] Huxley AF, Simmons RM (1971). Proposed mechanism of force generation in striated muscle.. Nature.

[2] Rayment I, Rypniewski WR, Schmidt-Base K, Smith R, Tomchick DR (1993). Threedimensional structure of myosin subfragment-1: a molecular motor.. Science.

[3] Rayment I, Holden HM, Whittaker M, Yohn CB, Lorenz M (1993). Structure of the actinmyosin complex and its implications for muscle contraction.. Science.

[4] Geeves M, Holmes K (1999). Structural mechanism of muscle contraction.. Annu Rev Biochem.

[5] Piazzesi G, Lombardi V (1995). A cross-bridge model that is able to explain mechanical and energetic properties of shortening muscle.. Biophys J.

[6] Hopkins SC, Sabido-David C, van der Heide UA, Ferguson RE, Brandmeier BD (2002). Orientation changes of the myosin light chain domain during filament sliding in active and rigor muscle.. J Mol Biol.

[7] Piazzesi G, Reconditi M, Linari M, Lucii L, Sun YB (2002). Mechanism of force generation by myosin heads in skeletal muscle.. Nature.

[8] Piazzesi G, Reconditi M, Linari M, Lucii L, Bianco P (2007). Skeletal muscle performance determined by modulation of number of myosin motors rather than motor force or stroke size.. Cell.

[9] Thomas DD, Kast D, Korman VL (2009). Site-directed spectroscopic probes of actomyosin structural dynamics.. Annu Rev Biophys.

[10] Kishino A, Yanagida T (1988). Force measurements by micromanipulation of a single actin filament by glass needles.. Nature.

[11] Finer JT, Simmons RM, Spudich JA (1994). Single myosin molecule mechanics: piconewton forces and nanometre steps.. Nature.

[12] Ishijima A, Doi T, Sakurada K, Yanagida T (1991). Sub-piconewton force fluctuations of actomyosin in vitro.. Nature.

[13] Molloy JE, Burns JE, Kendrick-Jones J, Tregear RT, White DC (1995). Movement and force produced by a single myosin head.. Nature.

[14] Kitamura K, Tokunaga M, Iwane AH, Yanagida T (1999). A single myosin head moves along an actin filament with regular steps of 5.3 nanometres.. Nature.

[15] Kitamura K, Tokunaga M, Esaki S, Iwane AH, Yanagida T (2005). Mechanism of muscle contraction based on stochastic properties of single actomyosin motors observed *in vitro*.. Biophysics.

[16] Pate E, Cooke R (1989). A model of crossbridge action: the effects of ATP, ADP and Pi.. J Muscle Res Cell Motil.

[17] Huxley AF, Tideswell S (1996). Filament compliance and tension transient in muscle.. J Muscle Res Cell Mot.

[18] Duke TA (1999). Molecular model of muscle contraction.. Proc Natl Acad Sci USA.

[19] Smith DA, Geeves MA, Sleep J, Mijailovich SM (2008). Towards a unified theory of muscle contraction. i: Foundations.. Ann Biomed Eng.

[20] Mansson A (2010). Actomyosin-ADP states, interhead cooperativity, and the force-velocity relation of skeletal muscle.. Biophys J.

[21] Marcucci L, Truskinovsky L (2010). Muscle contraction: A mechanical perspective.. Eur Phys J E Soft Matter.

[22] Magnasco OM (1993). Forced thermal ratchets.. Phys Rev Lett.

[23] Prost J, Chauwin JF, Peliti L, Ajdari A (1994). Asymmetric pumping of particles.. Phys Rev Lett.

[24] Iwaki M, Iwane AH, Shimokawa T, Cooke R, Yanagida T (2009). Brownian search-and-catch mechanism for myosin-VI steps.. Nat Chem Biol.

[25] Huxley AF (1957). Muscle structure and theories of contraction.. Prog Biophys Biophys Chem.

[26] Huxley HE (1969). The mechanism of muscular contraction.. Science.

[27] Spudich JA (1994). How molecular motors work.. Nature.

[28] Takano M, Terada TP, Sasai M (2010). Unidirectional Brownian motion observed in an in silico single molecule experiment of an actomyosin motor.. Proc Natl Acad Sci USA.

[29] Kaya M, Higuchi H (2010). Nonlinear elasticity and an 8-nm working stroke of single myosin molecules in myofilaments.. Science.

[30] Jülicher F, Ajdari A, Prost J (1997). Modeling molecular motors.. Rew Mod Phys.

[31] Howard J (2001). Mechanics of motor proteins and the cytoskeleton.. Sinauer, Sunderland, Ma.

[32] Marcucci L, Yanagida T (2011). Analysis of the dwell time in a bi-dimensional Brownian multistable system: an application to molecular motors.. submitted.

[33] Piazzesi G, Lucii L, Lombardi V (2002). The size and the speed of the working stroke of muscle myosin and its dependence on the force.. J Physiol.

[34] Gardiner C (2004). Handbook of Stochastic Methods: for Physics, Chemistry and the Natural Sciences (Springer Series in Synergetics).. Springer, 3rd edition.

[35] Piazzesi G, Lombardi V (1996). Simulation of the rapid regeneration of the actin-myosin working stroke with a tight coupling model of muscle contraction.. J Muscle Res Cell Motil.

[36] Hill AV (1938). The Heat of Shortening and the Dynamic Constraints of Muscle.. Proceedings of the Royal Society B.

[37] Parmeggiani A, Jülicher F, Ajdari A, Prost J (1999). Energy transduction of isothermal ratchets: generic aspects and specific examples close to and far from equilibrium.. Phys Rev E.

[38] Barclay CJ (1998). Estimation of cross-bridge stiffness from maximum thermodynamic efficiency.. J Muscle Res Cell Motil.

[39] Sekimoto K (1997). Kinetic characterization of heat bath and the energetics of thermal ratchet models.. Journal of The Physical Society of Japan.

[40] Kodera N, Yamamoto D, Ishikawa R, Ando T (2010). Video imaging of walking myosin V by high-speed atomic force microscopy.. Nature.

[41] Ford LE, Huxley AF, Simmons RM (1977). Tension responses to sudden length change in stimulated frog muscle fibres near slack length.. J Physiol (Lond).

[42] Reconditi M, Brunello E, Linari M, Bianco P, Narayanan T (2011). Motion of myosin head domains during activation and force development in skeletal muscle.. Proc Natl Acad Sci USA.

