# Supplementary material for: From Single Molecule Fluctuations to Muscle Contraction: A Brownian Model of A.F. Huxley's Hypotheses
Source: PLoS One. 2012 Jul 16;7(7):e40042. doi: 10.1371/journal.pone.0040042 (PMC3397984; doi:10.1371/journal.pone.0040042)
Supplement: Text S1 — (PDF) [file pone.0040042.s006.pdf]

Supporting Information text

Supporting Information Text

Supplementary Methods The motion of an over-damped particle thermally fluctuating in the system described in Fig. 1 of the main text is described by the stochastic ODE:

$$\eta_x \dot{x}_i = -\omega_i(t) E'_a(x_i) - E'_e(x_i, l_i) + \sqrt{\eta_x k_B \theta}\,\Gamma(t), \tag{S1}$$

with

$$\begin{cases} < \Gamma(t) >= 0 \\ < \Gamma(t_1)\Gamma(t_2) >= 2\delta(t_1 - t_2). \end{cases} \tag{S2}$$

Since we are interested both in steady state and transient skeletal muscle behaviour, we approach the system of equations by direct numerical simulation using the Euler-Maruyama method [1]. Defining two subsequent times separated by the time step τ_{step} as $t_{n+1} = t_n + \tau_{step}$, we recursively define $x_i(t_n)$ as:

$$x_i(t_{n+1}) = x_i(t_n) - \eta_x^{-1}[\omega_i(t_n) E'_a(x_i(t_n)) - E'_e(x_i(t_n), l_i)]\tau_{step} + \sqrt{\eta_x^{-1} k_B \theta} \int_{t_n}^{t_{n+1}} \Gamma(t). \tag{S3}$$

From the properties of Γ detailed in (S2), the integral in the last term is a random variable which is normally distributed with zero mean and variance τ_{step}. Therefore we can substitute it with the function $\sqrt{2\tau_{step}}\,w(0,1)$, where

$$\begin{cases} < w >= 0 \\ < w_1 w_2 >= \delta_{12}. \end{cases} \tag{S4}$$

Simulations were performed with Matlab® and $w(0,1)$ was generated by the built-in function *randn*. Data analysis and plots were also generated by Matlab®.

The populations of attached (A) and detached (D) myosin molecules are described by the reaction:

$$A \leftrightarrows^{k_+}_{k_-} D \tag{S5}$$

where k_+ and k_- are the first order rate constants described in Fig. 1 of the main text. At each time step the populations are updated with probabilities of switching from one state to the other:

$$\begin{cases} p_{A-D} = \dfrac{k_-}{k_+ + k_-}\left(1 - e^{-\tau_{step}(k_+ + k_-)}\right) \\ p_{D-A} = \dfrac{k_+}{k_+ + k_-}\left(1 - e^{-\tau_{step}(k_+ + k_-)}\right) \end{cases} \tag{S6}$$

to a random number generated by the built-in function *rand*.

References

1. Risken H (1996) The Fokker-Planck Equation: Methods of Solutions and Applications. Springer Series in Synergetics. Springer, 2nd ed. 1989. 3rd printing edition.

2. Marcucci L, Yanagida T (2011) Analysis of the dwell time in a bi-dimensional Brownian multi-stable system: an application to molecular motors. submitted .

3. Kitamura K, Tokunaga M, Esaki S, Iwane AH, Yanagida T (2005) Mechanism of muscle contraction based on stochastic properties of single actomyosin motors observed *in vitro*. Biophysics 1: 1-19.

4. Reconditi M, Brunello E, Linari M, Bianco P, Narayanan T, et al. (2011) Motion of myosin head domains during activation and force development in skeletal muscle. Proc Natl Acad Sci USA 108: 7236–7240.
